# Characterisation of Systemic Sclerosis in a Jordanian Cohort: Clinical Features, Diagnostic Patterns, and Health-Related Quality of Life

**DOI:** 10.31138/mjr.140326.jcs

**Published:** 2026-06-19

**Authors:** Fatima Alnaimat, Salameh Al-Halaseh, Jehad Feras AlSamhori, Suzan Hanandeh, Juman Shamaileh

**Affiliations:** 1 Department of Internal Medicine, Division of Rheumatology, School of Medicine, The University of Jordan, Amman, Jordan;; 2 School of Medicine, University of Jordan, Amman, Jordan;; 3 Jordan University Hospital, Amman, Jordan

**Keywords:** systemic scleroderma, Raynaud disease, quality of life, cross-sectional studies, disability evaluation, patient reported outcome measures

## Abstract

**Objective:**

To describe the clinical and demographic characteristics of systemic sclerosis (SSc) in a Jordanian cohort and assess patients’ psychological well-being, quality of life, and functional disability.

**Methods:**

This cross-sectional study included all consecutive SSc patients seen at a single rheumatology clinic at Jordan University Hospital, Amman, between January and October 2023. Demographic and clinical data were collected via a structured questionnaire. Depression was assessed with the PHQ-9, and fibromyalgia screening with the FiRST questionnaire. Disease activity was measured using the modified Rodnan Skin Score (mRSS) and Scleroderma Health Assessment Questionnaire (SHAQ), disability with the Cochin Hand Function Scale (CHFS), and quality of life with the SF-36.

**Results:**

Fifty-seven patients were enrolled; mean age 50.7 ± 12.9 years, 53 (93.0%) female. Thirty-two (56.1%) had limited SSc, and 25 (43.9%) had diffuse disease. Raynaud’s phenomenon was present in 49 (86.0%), digital ulcers in 28 (49.1%), and interstitial lung disease in 14 (24.6%). Pulmonary arterial hypertension was identified in 16 (28.1%). Microstomia was observed in 44 (77.2%) and gastroesophageal reflux in 21 (36.8%). Among 43 patients with DXA scans, 11 (19.3% of total cohort) had osteopenia and 7 (12.3%) osteoporosis.SF-36 physical and mental component scores were similarly impaired across subtypes. Only 1 patient (1.8%) met criteria for mild depression, and none met criteria for fibromyalgia.

**Conclusion:**

This is the first descriptive study of SSc in Jordan, highlighting a significant burden of organ involvement, comorbidities, and functional disability in this understudied population.

## INTRODUCTION

Systemic Sclerosis (SSc) is an autoimmune disease characterised by progressive fibrosis of the skin and internal organs, as well as vasculopathy. SSc is a rare multisystem autoimmune disease driven by vasculopathy, immune dysregulation, and progressive fibrosis, affecting approximately 233–277 cases per million.^1,2^ Since skin thickening is present in nearly all affected individuals, most commonly affecting the hands, the disease is categorised into two main clinical subtypes based on the pattern of skin involvement. In limited cutaneous (lc) SSc, skin involvement is confined to the face, hands, forearms, lower legs, and feet. In contrast, diffuse cutaneous (dc) SSc involves more extensive areas, including the upper arms, thighs, and trunk.^3^

Besides skin thickening, patients with SSc may experience various manifestations, including joint pain, fatigue, Raynaud’s phenomenon, and deep organ involvement such as the lungs, heart, and gastrointestinal system. These disease manifestations often lead to physical limitations, reduced mobility, and impaired functional abilities,^4^ which can hinder daily activities and negatively affect emotional well-being. SSc imposes a significant socioeconomic burden, with high rates of work disability and reduced productivity reported across populations.^5^ Visible disfigurement affecting the face and hands has been shown to impair body image, self-confidence, and quality of life, representing an underrecognised burden in SSc.^6^

Depressive symptoms are common in SSc patients; a study using the Beck Depression Inventory found that 51–65% of SSc patients exhibited depression.^7^ A single study found that 62% of patients with extensive skin involvement also had fibromyalgia symptoms, adding to the psychological toll that scleroderma has on its patients.^8^ Various scales have been used to estimate the burden of the disease on patients, such as the Scleroderma Health Assessment Questionnaire (SHAQ), which provides a disease-specific measure of functional limitations and overall well-being^4^ and the Cochin Hand Function Scale (CHFS).

Data on SSc from the Arab world remain scarce, with published cohorts limited to Qatar^9^ and the United Arab Emirates.^10^ No data have previously been published from Jordan or the broader Levant region. Given the genetic, environmental, and healthcare system differences across these populations, extrapolating findings from other regional or international registries may not fully capture the disease burden in Jordanian patients. This study therefore aims to provide an initial descriptive characterisation of SSc in a Jordanian cohort, including demographic and clinical features, serological profiles, treatment patterns, and patient-reported outcomes encompassing functional disability and health-related quality of life.

## METHODS

### Ethical approval

This study was approved by the Institutional Review Board of the University of Jordan, School of Medicine, and Jordan University Hospital (protocol number 10/2023/9360). All procedures followed the ethical standards of the responsible committee and the Declaration of Helsinki. Written informed consent was obtained from all participants prior to data collection.

### Patient recruitment

This study is a cross-sectional analysis of all consecutive patients diagnosed with SSc who visited a single-provider rheumatology clinic at Jordan University Hospital (JUH) in Amman, Jordan. Data collection spanned from January to October 2023. The study complied to the Strengthening the Reporting of Observational Studies in Epidemiology (STROBE) guidelines for cross-sectional studies.^11^

The inclusion criteria were all adult patients of both genders who fulfilled the 2013 American College of Rheumatology (ACR)/European League against Rheumatism (EULAR) classification criteria for SSc^12^ and were willing to provide their informed written consent. Patients who didn’t consent or didn’t complete their assessment were excluded. The patients were recruited during their regular clinic visits, with data collection and physical examinations conducted by two rheumatology fellows (SA, JA) after the completion of their clinical appointments. Metabolic parameters were collected from clinical records to describe comorbidities that may influence disease severity, and patient-reported outcomes. A structured questionnaire was used to collect demographic, clinical and laboratory data. Disease duration was defined as the time from the onset of the first SSc-related symptom to the date of enrolment. The time from the first non-Raynaud’s symptom to formal diagnosis was additionally recorded to assess potential diagnostic delay.

Continuous variables were expressed as median and interquartile range (IQR) or mean ± standard deviation (SD), while categorical variables were summarised as frequencies and percentages. The Shapiro-Wilk test was used to assess the normality of data distribution. Fisher’s exact test and the chi-square test were employed for categorical comparisons, whereas the Mann-Whitney U test and independent t-test were used for continuous comparisons. A p-value < 0.05 was considered statistically significant for all analyses.

### Assessment of Pulmonary Arterial Hypertension and Interstitial Lung Disease

Pulmonary arterial hypertension (PAH) was assessed using transthoracic echocardiography performed within one year of enrolment. PAH was defined as an estimated right ventricular systolic pressure (RVSP) greater than 40 mmHg or other echo findings suggestive of elevated pulmonary pressures. Interstitial lung disease (ILD) was diagnosed using a combination of pulmonary function tests (PFTs) and high-resolution computed tomography (HRCT). HRCT scans were reviewed by a consultant radiologist, and ILD was confirmed when interstitial abnormalities consistent with systemic sclerosis were present. PFT results were used to classify impairment according to the categories reported in the study tables. Forced vital capacity (FVC) and diffusing capacity for carbon monoxide (DLCO) were classified according to percent predicted values documented in clinical records. For DLCO, normal was defined as 80 percent or higher, mild reduction as 60–79 percent, moderate reduction as 40–59 percent, and severe reduction as less than 40 percent. For FVC, normal was 80 percent or higher, mild reduction 70–79 percent, moderate reduction 60–69 percent, moderately severe reduction 50–59 percent, and severe reduction less than 50 percent.

### Assessment of Skin thickening

The skin thickness was assessed using the modified Rodnan skin score (mRSS), a validated tool used as a primary or secondary outcome measure in clinical studies of systemic sclerosis.^13^ Skin thickness is measured as a proxy for disease activity, severity, and mortality in individuals with dcSSc.^13^ The score measures skin thickness in seventeen body areas such as hands, thighs and face. Each location receives a score ranging from 0 to 3, with 3 representing the most severe thickness and 0 indicating no thickness appreciated. There are three different ways to calculate the final score for each patient. We used the Maximum Score method, in which individual anatomical areas are scored based on the maximum severity of local involvement.^13^ Skin involvement severity was defined using the modified Rodnan Skin Score (mRSS), with mild disease defined as a score less than 10, moderate disease as 10–20, and severe disease as greater than 20.

### Measurement of the Psychological Well-being of the Patients

Depression was screened for using the PHQ-9 score, which is a novel tool for making criteria-based diagnoses of depression and other mental illnesses typically seen in primary care.^11^ This measure is a 9-item depression assessment from the full PHQ. Major depression is diagnosed if five or more symptoms are present for “more than half the days” in the past two weeks, including depressed mood or anhedonia. Other depression is diagnosed if 2 - 4 symptoms meet this frequency, also including either depressed mood or anhedonia. Suicidal thoughts count if present at any time, regardless of duration.^14^

Screening for fibromyalgia was evaluated using the FiRST score. This self-questionnaire covers six symptom domains in fibromyalgia, including pain, fatigue, non-painful abnormal sensations, functional somatic symptoms, sleep disturbances, and cognitive difficulties. The cut-off score of ≥5/6 achieved the highest sensitivity and specificity for the presence of fibromyalgia.^15^

### Measurement of Disability and Quality of Life

The Cochin Hand Function Scale (CHFS) was utilised to assess the functional disability of the patients. CHFS is a validated and straightforward questionnaire consisting of 18 questions that determine disability in daily activities. The score ranges from 0 to 90, with 90 indicating the maximum possible dysfunction. Its validity and reliability have been demonstrated specifically in SSc populations.^16^

The SHAQ-DI is a validated disease-specific extension of the HAQ, widely used to assess functional disability and health-related quality of life in SSc.^17^ The HAQ was created to provide a comprehensive measure of outcomes for patients with a wide range of rheumatic disorders, including but not limited to rheumatoid arthritis, osteoarthritis, and scleroderma.^18^ The Disability Index assesses eight categories: 1) arising, 2) dressing and grooming, 3) eating, 4) walking, 5) hygiene, 6) reach, 7) grip, and 8) common daily activities. For each of these categories, patients report the amount of difficulty they have in performing two or three specific activities. A combined SHAQ score of 0 to 1 indicates mild to moderate disability, a score of 1 to 2 indicates moderate to severe disability, and a score of 2 to 3 indicates severe to very severe disability.

The Short Form-36 (SF-36) is a self-administered tool that assesses health-related quality of life. The SF-36 was designed for use in clinical practice, research, health policy evaluation, and general population surveys.^19^ It encompasses eight distinct domains, including physical functioning, social functioning, and emotional well-being, ultimately providing a comprehensive physical and mental perspective on the patient’s health.^20^ Each of the 8 domains is scored independently on the 0–100 scale. There are different ways to score the SF-36.^20^ In our analysis, we averaged the domains and used the physical and mental component scores to obtain a broader view.

## RESULTS

Continuous variables were summarised as mean ± standard deviation or median (IQR), and categorical variables as frequencies and percentages. Group comparisons were performed using independent t tests, Mann–Whitney U tests, chi-squared tests, or Fisher’s exact tests according to variable distribution. Missing data were handled using complete case analysis with no imputation. Ninety-five percent confidence intervals were reported where relevant. A two-sided p-value less than 0.05 was considered statistically significant.

### Demographics of the study sample

Fifty-seven patients were enrolled in the study, with a mean age of 50.70 ± 12.86 years (mean ± standard deviation). The majority were females (52 patients, 93.0%), married (44 patients, 77.2%), unemployed (31 patients, 54.4%), non-smokers (47 patients, 82.5%), and had a secondary school education (26 patients, 45.6%). Four participants reported a positive family history of SSc (7.0%). **Table 1** summarises the sociodemographic and clinical characteristics of patients.

**Table 1.** Sociodemographic and clinical characteristics of systemic sclerosis patients stratified by disease subtype (limited vs. diffuse).

**Items Frequency (percentage)**	**Total (N=57)**	**Limited 32 (56.1%)**	**Diffuse 25 (43.9%)**	**P-value**
**Age in years ^A^**	50.70 ± 12.86	53.19 ± 12.54	47.52 ± 12.81	0.099
**Sex ^B^**				0.990
Male	4 (7.0)	2 (3.5)	2 (3.5)	
Female	53 (93.0)	30 (52.6)	23 (40.4)	
**Marital Status ^B^**				0.199
Married	44 (77.2)	26 (45.6)	18 (31.6)	
Divorced	1 (1.8)	1 (1.8)	0 (0.0)	
Widowed	4 (7.0)	3 (5.3)	1 (1.8)	
Single	8 (14.0)	2 (3.5)	6 (10.5)	
**Occupation ^B^**				0.328
White collar	7 (12.3)	2 (3.5)	5 (8.8)	
Blue collar	3 (5.3)	2 (3.5)	1 (1.8)	
Unemployed	31 (54.4)	20 (35.1)	11 (19.3)	
Retired	16 (28.1)	8 (14.0)	8 (14.0)	
**Education ^C^**				0.130
Elementary	15 (26.3)	11 (19.3)	4 (7.0)	
Secondary	26 (45.6)	15 (26.3)	11 (19.3)	
Undergraduate degree	16 (28.1)	6 (10.5)	10 (17.5)	
**Duration of disease in years ^D^**	5 [2 - 13]	6 [2 - 15.75]	5 [2 - 10.5]	0.424
**Smoking status B**				0.632
Non-Smoker	47 (82.5)	25 (43.9)	22 (38.6)	
Current Smoker	8 (14.0)	6 (10.5)	2 (3.5)	
Ex Smoker	2 (3.5)	1 (1.8)	1 (1.8)	
**Positive past medical history ^C^**				
HTN	14 (24.6)	8 (14.0)	6 (10.6)	0.931
DM	4 (7.0)	2 (3.5)	2 (3.5)	0.797
HLD	1 (1.8)	1 (1.8)	0 (0.0)	0.373
IHD	4 (7.1)	3 (5.3)	1 (1.8)	0.431
Cancer	1 (1.8)	1 (1.8)	0 (0.0)	0.373
Thyroid diseases	9 (15.8)	5 (9.4)	4 (7.5)	0.969
**BMI ^A^**	25.17 ± 5.16	25.12 ± 5.4	25.22 ± 4.97	0.946
**Family history of SSc ^B^**				0.797
Negative history	53 (93.0)	30 (52.6)	23 (40.4)	
Positive history	4 (7.0)	2 (3.5)	2 (3.5)	

BMI: body mass index; DM: diabetes mellitus; HTN: hypertension; HLD: hyperlipidaemia; IHD: ischemic heart disease; kg: kilogram; m: metre.

A: Independent t-test; b: Fisher's Exact Test; c: Chi-squared test; d: Mann–Whitney U test. Categorical variables are represented by the number (%) of the overall patients while continues variables are represented by mean ± standard deviation or median (IQR) as appropriate. Bold values mean they are statistically significant (P value < 0.05).

### Prevalence of comorbidities among the study sample

**Table 1** presents the comorbidities among the study sample. The most reported conditions were hypertension (14 patients, 24.6%), thyroid disease (9 patients, 15.8%), and diabetes mellitus (4 patients, 7.0%). The mean BMI was 25.17 ± 5.16 m^2^, and most participants had blood pressure readings within the normal range, with a mean systolic pressure of 116.55 ± 13.01 mmHg and a mean diastolic pressure of 72.35 ± 10.12 mmHg. Metabolic and cardiovascular comorbidities available in clinical records were summarised descriptively to provide context about overall patient health (**Table 2**). The mean HDL cholesterol was 54.23 ± 17.63 mg/dL, LDL was 106.07 ± 40.72 mg/dL, and total cholesterol (TC) was 173.54 ± 48.31 mg/dL. The mean TC/HDL ratio was 3.41 ± 1.27, suggesting an average moderate cardiovascular risk (**Table 2**). The average HbA1c was 5.91 ± 1.31%.

**Table 2.** Laboratory investigations for study patients (n = 57).

**Variable**	**Total**	**Limited (%)**	**Diffuse (%)**	**P-value**
**DEXA scan ^B^**				0.269
Normal Z score	8 (14.0)	6 (10.5)	2 (3.5)	
Low Z score	12 (21.1)	6 (10.5)	6 (10.5)	
Normal T score	5 (8.8)	1 (1.8)	4 (7.0)	
Osteopenia	11 (19.3)	6 (10.5)	5 (8.8)	
Osteoporosis	7 (12.3)	6 (10.5)	1 (1.8)	
**Hgb ^A^**	12.50 ± 1.90	12.44 ± 1.81	12.57 ± 2.03	0.802
**WBC** ^A^	7.49 ± 2.64	7.23 ± 2.86	7.85 ± 2.34	0.387
**PLT ^A^**	269.71 ± 94.91	247.22 ± 90.76	299.71 ± 93.90	**0.039**
**Vitamin D** ^A^	39.09 ± 15.92	37.20 ± 14.59	41.60 ± 18.63	0.628
**LDL ^A^**	106. 07 ± 40.72	106.96 ± 40.61	104.86 ± 41.88	0.863
**HDL ^A^**	54.23 ± 17.63	57.39 ± 17.30	49.97 ± 17.59	0.156
**TC ^A^**	173.54 ± 48.31	170.59 ± 47.04	177.74 ± 51.04	0.627
**TG ^A^**	153.1 ± 94.7	134.19 ± 65.15	165.19 ± 112.92	0.241
**TC / HDL ^A^**	3.41 ± 1.27	3.20 ± 1.08	3.71 ± 1.50	0.189
**HbA1c ^A^**	5.91 ± 1.31	5.87 ± 1.18	5.96 ± 1.49	0.819
**TSH ^A^**	2.62 ± 1.64	2.15 ± 1.42	3.21 ± 1.73	**0.020**
**Cr ^A^**	0.64 ± 0.18	0.68 ± 0.21	0.59 ± 0.13	0.083
**Albumin ^A^**	4.38 ± 1.90	4.23 ± 0.48	4.59 ± 1.73	0.267
**ALT ^A^**	20.94 ± 18.84	22.55 ± 24.05	18.79 ± 7.76	0.464
**AST ^A^**	20.26 ± 9.54	21.41 ± 11.46	18.73 ± 6.04	0.303
**GGT ^A^**	21.66 ± 21.03	21.31 ± 23.19	22.08 ± 18.69	0.899

ALT: alanine aminotransferase; AST: aspartate aminotransferase; Cr: creatinine; DEXA: dual-energy X-ray absorptiometry; GGT: gamma-glutamyl transferase; PLT: platelets; TC: total cholesterol); TC/HDL: total cholesterol to high-density lipoprotein ratio; TG: triglycerides; TSH: thyroid-stimulating hormone; WBC: white blood cells.

A: Independent t-test; b: Fisher's Exact Test; c: Chi-squared test; d: Mann-Whitney U test. Categorical variables are represented by the number (%) of the overall patients while continues variables are represented by mean ± standard deviation or median (IQR) as appropriate. Bold values mean they are statistically significant (P value < 0.05).

Echocardiographic data collected within the past year of enrolment were available for all the patients. Among them, 23 patients (40.4%) had normal findings. Cardiomyopathy with a reduced left ventricular ejection fraction (<50%) was observed in 6 patients (10.5%). Valvular heart disease was present in 18 patients (31.6%), while left ventricular hypertrophy was identified in 10 patients (17.5%). Additionally, pulmonary arterial hypertension (PAH) was reported in a total of 16 patients (28.1%), among whom 12 patients were of the diffuse systemic sclerosis group and 4 patients were from the limited disease group.

As for the patients’ bone health, Dual-energy X-ray Absorptiometry (DXA scan) results were available for 43 patients (75.4%). Among them, 13 patients (30.2%) had normal bone density, with 8 patients (14.0%) showing normal Z-scores and 5 patients (8.8%) showing normal T-scores, as determined by age-appropriate assessment. Low bone density findings included low Z-scores in 12 patients (21.1%), osteopenia in 11 patients (19.3%), and osteoporosis in 7 patients (12.3%).

### Clinical Characteristics of Systemic Sclerosis in the Study Sample

The median disease duration was 5 years (The Interquartile Range [IQR] 2 to 13), and the age of onset had a median of 38 years, with a range from 33 to 53 years. Thirty-two participants had lc SSc (56.1%), while the remaining had dc SSc (43.9%). The most reported initial symptom prior to the diagnosis of SSc was Raynaud’s phenomenon, observed in 32 patients (56.1%), followed by generalised weakness in 13 patients (22.8%) and arthralgia in 10 patients (17.5%). Raynaud’s phenomenon was ultimately present in 49 patients (86.0%). Digital ulcers were observed in 28 patients (49.1%), telangiectasia in 23 patients (40.4%), and calcinosis was present in 10% of the cohort. Microstomia was identified in 44 patients (77.2%), xerostomia in 23 (40.4%), dysphagia in 19 (33.3%), and gastroesophageal reflux disease (GERD) in 21 (36.8%). Arthralgia was noted in 42 patients (73.7%). Among respiratory symptoms, cough was the most frequently reported (21 patients, 36.8%), followed by shortness of breath (13 patients, 22.8%). Interstitial lung disease was confirmed in 14 patients (24.6%). **Table 3** details the disease characteristics of the study sample.

**Table 3.** Disease characteristics of the study sample.

**Variable**	**Total**	**Limited (%)**	**Diffuse (%)**	**P-value**
**First reported symptom** ^B^				0.191
Raynaud's	32 (56.1)	17 (29.8)	15 (26.3)	
Generalised weakness	13 (22.8)	7 (12.3)	6 (10.5)	
Arthralgia	10 (17.5)	8 (14.0)	2 (3.5)	
MSK symptoms ^C^^*^				
Arthralgia	42 (73.7)	19 (33.3)	23 (40.4)	**0.006**
Muscle weakness	14 (24.5)	6 (10.5)	8 (14.0)	0.249
Joint contractures	11 (19.3)	7 (12.3)	4 (7.0)	0.577
Tendon friction rubs	2 (3.5)	1 (1.8)	1 (1.8)	0.990
Arthritis	1 (1.8)	1 (1.8)	0 (0.0)	0.990
Respiratory symptoms ^C^^*^				
ILD	14 (24.6)	3 (5.3)	11 (19.3)	0.003
Cough	21 (36.8)	11 (19.3)	10 (17.5)	0.662
SOB	13 (22.8)	6 (10.5)	7 (12.3)	0.409
Vascular symptoms ^C^^*^				
Raynaud phenomenon	49 (86.0)	27 (47.4)	22 (38.6)	0.696
Fingertip ulceration	28 (49.1)	18 (31.6)	10 (17.5)	0.223
Loss of finger pad	16 (28.1)	11 (19.3)	5 (8.8)	0.231
Telangiectasia	23 (40.4)	12 (21.1)	11 (19.3)	0.620
CVA	2 (3.5)	1 (1.8)	1 (1.8)	0.990
GI symptoms ^C^^*^				
Dysphagia	19 (33.3)	10 (17.5)	9 (15.8)	0.706
GERD	21 (36.8)	14 (24.6)	7 (12.2)	0.221
Gastroparesis	1 (1.8)	1 (1.8)	0 (0.0)	0.990
Altered bowel habits	14 (24.6)	9 (15.8)	5 (8.8)	0.479
Miscellaneous features ^C^^*^				
Microstomia	44 (77.2)	22 (38.6)	22 (38.6)	0.086
Xerostomia	23 (40.4)	11 (19.3)	12 (21.1)	0.298
Calcinosis	10 (17.5)	5 (8.8)	5 (8.8)	0.667
**DLCO** ^B^				0.370
Normal	18 (31.6)	13 (23.6)	5 (9.1)	
Mild	15 (26.3)	9 (16.4)	6 (10.9)	
Moderate	13 (22.8)	5 (9.1)	8 (14.5)	
Severe reduction	4 (7.0)	2 (3.6)	2 (3.6)	
**FVC** ^B^				0.151
Normal	20 (35.1)	14 (25.5)	6 (10.9)	
Mild	19 (33.3)	12 (21.8)	7 (12.7)	
Moderate	8 (14.0)	3 (5.5)	5 (9.1)	
Moderately severe	3 (5.3)	1 (1.8)	2 (3.6)	
Severe	3 (5.3)	0 (0.0)	3 (5.5)	
Very severe	0 (0.0)	0 (0.0)	0 (0.0)	
**FEV1** ^B^				0.289
Normal	29 (50.9)	20 (36.4)	9 (16.4)	
Mild	10 (17.5)	4 (7.3)	6 (10.9)	
Moderate	6 (10.5)	3 (5.5)	3 (5.5)	
Moderately severe	5 (8.8)	2 (3.6)	3 (5.5)	
Severe	4 (7.0)	1 (1.8)	3 (5.5)	
Very severe	1 (1.8)	1 (1.8)	0 (0.0)	
**FEV1/FVC** ^C^				0.278
>80	26 (45.6)	17 (30.9)	9 (16.4)	
<80	29 (50.9)	14 (25.5)	15 (27.3)	
Echo ^C^^*^				0.475
LVH	10 (17.5)	6 (11.5)	4 (7.7)	
LVEF < 50%	6 (10.5)	3 (5.8)	3 (5.8)	
Valvular disease	18 (31.6)	12 (21.1)	6 (10.5)	
PAH	16 (28.1)	12 (23.1)	4 (7.7)	
Pericardial effusion	2 (3.5)	2 (3.8)	0 (0.0)	
HRCT ^C^^*^				0.355
Reticulonodular infiltration	8 (14.0)	3 (4.2)	5 (7.0)	
Pleural thickening	2 (3.5)	1 (1.4)	1 (1.4)	
Fibrosis	23 (40.4)	12 (16.9)	11 (15.5)	
Honeycombing	16 (28.1)	4 (5.6)	12 (16.9)	
Intralobular septal thickening	2 (3.5)	1 (1.4)	1 (1.4)	
Emphysema	4 (7.0)	2 (2.8)	2 (2.8)	
Bronchiectasis	16 (28.1)	8 (11.3)	8 (11.3)	
Serological profile ^C^^*^				
ANA	35 (61.4)	21 (36.8)	14 (24.6)	0.459
Scl-70	34 (59.6)	14 (24.6)	20 (35.1)	0.006
Centromere	10 (17.5)	9 (15.8)	1 (1.8)	0.017
SSA	8 (14.0)	5 (8.8)	3 (5.3)	0.725
Anti-U1RNP	5 (8.8)	4 (4.2)	1 (1.0)	
SSB	4 (7.0)	3 (5.3)	1 (1.8)	0.623
				
ESR ^A^	31.52 ± 20.36	28.31 ± 18.64	35.79 ± 22.19	0.176
CRP ^A^	7.46 ± 8.79	6.67 ± 10.21	8.11 ± 6.61	0.637
**Raynaud's Drugs** ^B^				0.247
No drug	11 (19.3)	4 (7.0)	7 (12.3)	
Amlodipine/Nifedipine	45 (78.9)	27 (47.4)	18 (31.6)	
Sildenafil	1 (1.8)	1 (1.8)	0 (0.0)	
Current medications ^C^^*^				0.627
Mycophenolate	33 (57.9)	15 (26.3)	18 (31.6)	0.057
Prednisone	28 (49.1)	12 (21.1)	16 (28.1)	**0.047**
Hydroxychloroquine	20 (35.1)	11 (19.3)	9 (15.8)	0.898
Nintedanib	11 (19.3)	4 (7.0)	7 (12.3)	0.141
Mycophenolate + MTX	4 (7.0)	2 (3.5)	2 (3.5)	0.797
Rituximab	3 (5.3)	0 (0.0)	3 (5.3)	**0.044**
Cyclosporine	2 (3.5)	1 (1.8)	1 (1.8)	0.999
MTX alone	2 (3.5)	0 (0.0)	2 (3.5)	0.188
**Rodnan score** ^D^	10 [6 - 16.5]	8 [4 - 14]	14 [6.25 - 18]	0.112
**Rodnan score interpretation** ^B^				0.351
Mild skin involvement	40 (70.2)	25 (43.9)	15 (26.3)	
Moderate skin involvement	12 (21.0)	5 (8.8)	7 (12.3)	
Severe skin involvement	5 (8.8)	2 (3.5)	3 (5.3)	

ANA: antinuclear antibody; Anti-U1RNP: anti-U1 ribonucleoprotein; Centromere: anti-centromere antibody; CRP: C-reactive protein; CVA: cerebrovascular accident; DLCO: diffusing capacity of the lungs for carbon monoxide; ESR: erythrocyte sedimentation rate; FEV1: forced expiratory volume in 1 second; FVC: forced vital capacity; GERD: gastroesophageal reflux disease; GI: gastrointestinal; ILD: interstitial lung disease; LVEF: left ventricular ejection fraction; LVH: left ventricular hypertrophy; MSK: musculoskeletal; MTX: methotrexate; PAH: pulmonary arterial hypertension; Scl-70: anti-topoisomerase I antibody; SOB: shortness of breath; SSA: anti-Sjögren's-syndrome-related antigen A; SSB: anti-Sjögren's-syndrome-related antigen B.

A: Independent t-test; b: Fisher's Exact Test; c: Chi-squared test; d: Mann-Whitney U test. Categorical variables are represented by the number (%) of the overall patients while continues variables are represented by mean ± standard deviation or median (IQR) as appropriate. Bold values mean they are statistically significant (P value < 0.05).

* stands for multiple sets question.

There were no significant differences in disease duration among lc SSc patients with microstomia (median = 6.5 years, range 2–18), calcinosis (median = 8 years, range 2–29), and joint contractures (median = 13 years, range 4.5–16). However, among patients with dc SSc, those with calcinosis had significantly longer disease duration than those without (11 vs. 4 years, p=0.024). In contrast, no significant differences in disease duration were observed when comparing patients with and without microstomia (median = 5.5 years, range 3–10 vs. unaffected) or joint contractures (median = 8.5 years, range 6.5–10.5 vs. unaffected).

The serological profile of the patients showed that ANA was positive in 35 patients (61.4%), Scl-70 was positive in 34 patients (59.6%), while anti-centromere antibodies were present in 10 patients (17.5%). **Table 3** presents the details of disease characteristics of the study sample.

The mean erythrocyte sedimentation rate (ESR) at time of enrolment was 31.52 ± 20.36 mm/hr, while the mean C-reactive protein (CRP)level was 7.46 ± 8.79 mg/L. The mean Rodnan skin score was 10, with an IQR of 6 to 16.5, with no differences observed between limited (8 [4 - 14]) and diffuse (14 [6.25 - 18]) subsets. Most patients had mild skin involvement (70.2%), while moderate (21.0%) and severe (8.8%) skin involvement were less frequent.

### Pharmacotherapy for SSc among the study sample

Raynaud’s phenomenon was managed with either amlodipine or nifedipine in 45 patients (78.9%), while sildenafil was prescribed for one patient (1.8%). Mycophenolate was the most used immunosuppressive agent, prescribed to 33 patients (57.9%), followed by prednisolone in 28 patients (49.1%) and hydroxychloroquine in 20 patients (35.1%). A combination of mycophenolate and methotrexate (MTX) was used in 4 patients (7.0%). Rituximab was administered to 3 patients (5.3%), all of whom had the diffuse cutaneous subtype. The antifibrotic agent Nintedanib was prescribed to 11 patients with ILD (19.3%).

### Clinical and serological characteristics of the Patients by disease subtype

Significant differences were observed between patients with limited and diffuse systemic sclerosis in several clinical and treatment-related variables. Prednisolone was more frequently used in the diffuse subtype (p = 0.047), and Rituximab was exclusively prescribed to patients with the diffuse form (p = 0.044). Arthralgia was more common in the limited subtype (p = 0.006), whereas interstitial lung disease (ILD) was predominantly observed in the diffuse subtype (p = 0.003). Anti-Scl-70 antibodies were more frequently present in patients with the diffuse form (p = 0.006), whereas anti-centromere antibodies were more common in those with the limited subtype (p = 0.017).

No significant difference was found in the prevalence of calcinosis between the limited (17.5%) and diffuse (8.8%) subtypes (p = 0.667). Dysphagia was reported in 33.3% of patients, with similar prevalence in both limited (17.5%) and diffuse (15.8%) groups (p = 0.706). Gastroesophageal reflux disease (GERD) was observed in 36.8% of patients, more frequently in the limited subtype (24.6%) than in the diffuse subtype (12.2%), although the difference was not statistically significant (p = 0.221).

### Disease Impact on the Patients and Quality of Life Measures

The median score on the Cochin Hand Function Scale was 0 (IQR: 0–26), with 7 patients (12.5%) experiencing severe functional impairment. For the Scleroderma Health Assessment Questionnaire (SHAQ), the median score was 0.25 (IQR: 0–1), with severe disability reported by only a small proportion of participants (8.9%).

In terms of quality of life, assessed using the SF-36, patients with the limited subtype had a median score of 90 (IQR: 40–100), while those with the diffuse subtype had a median score of 85 (IQR: 52.5–100). There were no statistically significant differences between the median or mean SF-36 scores of the two subtypes (p > 0.05). The mean general health score was 55.48 ± 24.78 for patients with limited disease and 65.00 ± 21.82 for those with diffuse disease. The mean mental component score was similar between groups: 44.75 ± 9.89 for the limited type and 44.93 ± 11.22 for the diffuse type. Regarding mental health and fibromyalgia screening, depression was largely absent among participants. The PHQ-9 median score was 1 (IQR: 0–2), with only one patient (1.8%) falling into the mild depression category. Likewise, the FiRST score had a median of 0, indicating that none of the participants were likely to have fibromyalgia. **Table 4** provides a summary of the quality-of-life assessment scores across the study sample.

**Table 4.** Patient reported outcomes and functional measures in systemic sclerosis patients (n = 57).

**Variable**	**Total**	**Limited (%)**	**Diffuse (%)**	**P-value**
**PHQ-9 score ^D^**	1 [0 - 2]	1 [0 - 2]	1 [1 - 2]	0.193
**PHQ score interpretation ^B^**				0.990
Minimal or no depression	55 (98.2)	30 (53.6)	25 (44.6)	
Mild depression	1 (1.8)	1 (1.8)	0 (0.0)	
**FiRST score ^D^**	0 [0 - 0]	0 [0 - 0]	0 [0 - 0]	0.341
FiRST score interpretation				N/A
Unlikely fibromyalgia	56 (100.0)	31 (55.4)	25 (44.6)	
Likely fibromyalgia	0 (0.0)	0 (0.0)	0 (0.0)	
**Cochin Hand Function ^D^**	0 [0 - 26]	0 [0 - 12]	5.5 [0 - 40.75]	0.344
**Cochin scale interpretation ^B^**				0.340
No functional impairment	28 (50.0)	17 (30.4)	11 (19.6)	
Mild to moderate functional impairment	21 (37.5)	12 (21.4)	9 (16.1)	
Severe functional impairment	7 (12.5)	2 (3.6)	5 (8.9)	
**SHAQ score ^D^**	0.25 [0 - 1]	0.125 [0 - 1]	0.3125 [0 - 1.375]	0.284
**SHAQ score interpretation ^B^**				0.544
Mild disability	45 (80.4)	26 (46.5)	19 (33.9)	
Moderate disability	6 (10.7)	2 (3.6)	4 (7.1)	
Severe disability	5 (8.9)	3 (5.4)	2 (3.6)	
**SF-36 subscales**				
**Physical functioning ^D^**	85 [50 - 100]	90 [40 - 100]	85 [52.5 - 100]	0.463
**Role limitations due to physical health ^D^**	75 [0 - 100]	75 [0 - 100]	75 [0 - 100]	0.671
**Role limitations due to emotional problems ^D^**	66.7 [0 - 100]	66.7 [0 - 100]	66.7 [0 - 100]	0.674
**Energy/fatigue ^D^**	55 [45 - 65]	55 [50 - 65]	55 [40 - 65]	0.827
**Emotional wellbeing ^A^**	57.45 ± 19.07	59.35 ± 18.82	55.00 ± 19.50	0.406
**Social functioning ^D^**	75 [50 - 87.5]	62.5 [37.5 - 87.5]	87.5 [62.5 - 87.5]	0.072
**Pain ^D^**	70 [45 - 100]	70 [45 - 100]	68.75 [45 - 97.5]	0.762
**General Health ^A^**	59.64 ± 23.80	55.48 ± 24.78	65.00 ± 21.82	0.143
**Health change ^D^**	50 [25 - 75]	50 [25 - 75]	50 [50 - 75]	0.588
**Physical Component ^D^**	41.52 [29.61 - 43.62]	41.22 [24.42 - 44.36]	41.59 [33.33 - 43.34]	0.859
**Mental Component ^A^**	44.83 ± 10.39	44.75 ± 9.89	44.93 ± 11.22	0.950

PHQ-9: Patient Health Questionnaire-9; FiRST: Fibromyalgia Rapid Screening Tool; SHAQ: Scleroderma Health Assessment Questionnaire; SF-36: Short Form-36.

A: Independent t-test, b: Fisher's Exact Test, c: Chi-squared test, d: Mann–Whitney U test. Categorical variables are represented by the number (%) of the overall patients while continuous variables are represented by mean ± standard deviation or median (IQR) as appropriate. Bold values mean they are statistically significant (P value < 0.05).

## DISCUSSION

This study provides the first descriptive characterisation of systemic sclerosis in a Jordanian cohort, addressing a significant gap in the literature on SSc in the Arab world. While cohort studies have been published from Qatar,^9^ the United Arab Emirates,^10^ and other Middle Eastern and Asian countries,^21^ no prior data existed from Jordan or the broader Levant region. Compared to these regional cohorts, our study uniquely combines clinical and serological profiling with a comprehensive assessment of patient-reported outcomes including functional disability, hand function, health-related quality of life, depression screening, and fibromyalgia screening in the same population. The high prevalence of limited SSc (56.1%), the predominance of mycophenolate as the primary immunosuppressive agent, and the relatively low burden of functional disability compared to other published cohorts^22,23^ together represent novel observations from this underrepresented region. Because the sample size and subgroup numbers were limited, the analyses were exploratory and should be considered descriptive observations rather than confirmatory evidence.

In our cohort, the mean age at disease onset was 41.9 years, which is consistent with reports from European^24^ and Spanish populations.^25^ In contrast, a Qatari study found a significantly younger mean age of onset (34.5 years), though nearly half of that cohort consisted of non-Arab patients of mixed ethnicities.^9^ The strong female predominance observed in our cohort (93%) is consistent with the established epidemiology of SSc, with women to men ratios ranging from 4:1 to 10:1 reported across populations.^26,27^ This current study identified a greater frequency of the limited subtype of systemic sclerosis, which does not contraindicate the reported literature^21^ in which the limited subtype is the more common form of the disease. However, some parts of the world, such as the United Arab Emirates, Taiwan, China, East Malaysia and Singapore report a higher rate of dSSc.^10,21^

The serological profile of our cohort showed a high Scl-70 positivity rate of 59.6%, predominantly in diffuse SSc (p=0.006), exceeding rates reported in the Qatari,^9^ Malaysian,^21^ and UAE10 cohorts. ACA positivity was observed in 17.5%, predominantly in limited SSc (p=0.017), consistent with established serological-clinical associations. Scl-70 positivity carries prognostic significance, being associated with ILD development and progressive lung disease, while ACA positivity is linked to higher PAH risk but lower ILD risk.^28^

In our cohort, four participants (7.0%) reported a positive family history of SSc. Familial clustering of SSc has been well-documented, with evidence showing that the risk of SSc and other autoimmune diseases is increased among relatives of affected individuals, and that family factors account for more than two-thirds of the disease’s phenotypic variance.^29^ SSc is substantially more common in families with scleroderma (1.6%) compared to the general population (0.026%), making a positive family history a significant established risk factor for the disease. However, the absolute risk for each family member remains relatively small (<1%).^30^ In our cohort, the mean BMI was similar between limited and diffuse SSc subtypes (25.12 ± 5.40 kg/m^2^ vs. 25.22 ± 4.97 kg/m^2^, p = 0.946), indicating that it did not differ significantly by disease subset. While BMI did not differ significantly by disease subset, adipose tissue-derived adipokines have been implicated in SSc pathogenesis, and BMI has been identified as an independent factor associated with survival in SSc.^31–33^

Hypertension (HTN) was present in 14 patients (24.6%) in our cohort, aligning with previous studies reporting prevalence rates between 26.7% and 41.7%.^34,35^ Vascular changes inherent to SSc may predispose to hypertension through early microvascular and macrovascular dysfunction, with subsequent renal and cardiac involvement.^36^ Compared to the general population, those with systemic sclerosis have more than twice the risk of cardiovascular events.^37,38^ Type 2 diabetes mellitus (T2D) was found in 4 patients (7.0%) in our cohort, closely mirroring recent reports of 7.6% prevalence in SSc.^39^ Larger epidemiologic studies suggest that T2D prevalence is lower in SSc compared to the general population, possibly due to severe gastrointestinal involvement, malabsorption, and malnutrition, reducing obesity-related insulin resistance—a major driver of T2D.^39^

Raynaud’s phenomenon (RP) was among the most common initial presentation in our study, affecting 56.1% of patients. RP results from microangiopathy and dysregulation of microvascular tone, and it develops in nearly all patients with systemic sclerosis (95– 96%) during the disease course.^40,41^ As one of the most frequent causes of secondary RP, systemic sclerosis is often preceded by years of RP before other clinical features emerge.^42^ Notably, our study also identified a subgroup presenting alternative initial symptoms, such as weakness and arthralgia. Data from two large US cohorts showed that over 30% of patients presented without RP, often with puffy fingers, and were more likely to have severe disease and RNA polymerase III positivity.^43^ However, identifying secondary RP early and accurately is crucial, as it can speed up diagnosis and improve prognosis.^42^

In our cohort, most patients were treated with dihydropyridine calcium channel blockers (CCBs), such as amlodipine or nifedipine, with 45 patients (78.9%) receiving them. These medications have been shown to reduce overall patient disability by 0.4 points on a 10-cm visual analogue scale, as well as decrease the frequency and duration of RP attacks.^44^ One patient (1.8%) received sildenafil, a phosphodiesterase-5 inhibitor (PDE5i), while no patients required intravenous iloprost or fluoxetine. These patterns reflect the 2023 EULAR recommendations, which continue to support CCBs as first-line therapy for RP in SSc due to their proven effectiveness and safety.^45^

The treatment pattern observed in our cohort is consistent with real-world SSc practice reported in the literature. CCBs represent the most frequently prescribed vasoactive agent in real-world SSc cohorts, administered in up to 68% of patients across registries.^46^ The predominance of mycophenolate as the most used immunosuppressive agent in our cohort (57.9%) aligns with its current status as the preferred first-line immunosuppressant for SSc-related skin and lung involvement, as endorsed by the 2023 EULAR recommendations.^45^ Hydroxychloroquine was prescribed in 35.1% of our patients, reflecting its common use for constitutional and articular symptoms. Rituximab was exclusively used in diffuse SSc patients (5.3%), consistent with its reserved role for refractory disease.^45^

Arthralgia has been reported in approximately 70% of patients,^47^ aligning with our findings, where 42 patients (73.7%) reported joint pain. In contrast to Ostojić et al.,^48^ who reported arthralgia to be more prevalent in diffuse SSc, our cohort showed a significantly higher prevalence in the limited subtype (p=0.006), consistent with the Pakistani cohort.^49^ Joint pain may precede diagnosis in 20% of patients, present at diagnosis in 30%, and occur after diagnosis in 50%.^50^ Joint contractures lack a standardised definition, and reported prevalence varies.^47^ In our sample, 19.3% reported joint contractures. A three-year study by Bálint Z et al.^51^ found contractures were more frequent in dcSSc patients with elevated inflammatory markers and greater skin thickening. The prevalence of muscle weakness in a study was 22.8%,^52^ whereas in our study, it was 24.9%. For each increase in muscle severity score, the median HAQ-DI score increased, reflecting higher levels of disability.^52^

In both the limited and diffuse systemic sclerosis populations, gastrointestinal symptoms were prevalent. Compared to the limited dSSc group, twice as many patients reported symptoms of gastroesophageal reflux disease (GERD) in the dSSc group. This observation fits into previous studies.^21,53^ The oesophagus is the most frequently affected gastrointestinal organ in systemic sclerosis, with gastroesophageal reflux disease (GERD) being the predominant complication.^54^ GERD is often troublesome and significantly impacts quality of life, as shown in prior studies.^55^ Proton pump inhibitors remain the cornerstone of management.^56^ Patients tend to under-report gastrointestinal symptoms, even though they can be extremely bothersome and easily managed.^53^

Interstitial lung disease (ILD) was present in 14 patients (24.6%). Reported prevalence rates vary widely across populations. The prevalence of SSc-ILD is highly variable was estimated to be 56% in east Asia^57^ and was found to be 78.9% in a Pakistani cohort, where ILD was more common in diffuse cutaneous SSc than in limited cutaneous SSc.^49^ Our observed prevalence is lower than that reported in these studies, which may reflect differences in disease subtype distribution. In most patients with SSc who develop ILD, lung involvement occurs within the first three years of diagnosis, and 40–75% demonstrate reduced pulmonary function.^58^

In our cohort, 11 patients (19.3%) were treated with the antifibrotic agent Nintedanib. This intracellular tyrosine kinase inhibitor has been approved for idiopathic pulmonary fibrosis. It demonstrated efficacy in slowing forced vital capacity (FVC) decline in SSc-associated ILD.^59^ While generally reserved for progressive disease, there is ongoing debate about earlier initiation due to its proven benefit in reducing FVC decline.^60^ Real-world data from a recent observational study in a Greek population confirm the treatment response and acceptable safety profile of nintedanib in CTD-ILD, including SSc-ILD, supporting its use in routine clinical practice.^61^

Pulmonary arterial hypertension was identified in 16 patients (28.1%) in our cohort based on echocardiographic criteria (RVSP >40 mmHg). This figure is higher than the pooled prevalence of 6.4% reported in a meta-analysis of 24 studies using right heart catheterisation (RHC) as the reference standard,^62^ and higher than the 7% echocardiography-based prevalence reported in the Australian Scleroderma Cohort Study.^63^ The higher prevalence observed in our cohort likely reflects the use of echocardiography rather than RHC for PAH diagnosis, a well-recognised limitation that tends to overestimate true PAH prevalence due to the lower specificity of echocardiographic RVSP estimates. Additionally, the higher proportion of diffuse SSc in our PAH-positive patients (12 of 16, 75%) and the predominance of Scl-70 positivity in our cohort may have contributed to this finding, as diffuse SSc and Scl-70 positivity are associated with more severe cardiopulmonary involvement. These findings underscore the importance of confirming echocardiographically suspected PAH with RHC in future studies from this region. Primary myocardial involvement in SSc encompasses a broad spectrum of pathophysiology and clinical manifestations, and advances in cardiac imaging are improving our ability to detect subclinical disease early in this population.^64^ Valvular heart disease was also identified in 18 patients (31.6%) in our cohort, a finding consistent with the growing recognition of significant valvular pathology in SSc, including cases severe enough to require advanced interventions such as transcatheter aortic valve replacement.^65^

A single individual in our cohort with dSSc was positive for anticentromere antibodies (ACA), which are often found in the limited cutaneous subtype. A recent systematic review found that ILD, pulmonary hypertension, and scleroderma renal crisis were more common in ACA-dcSSc compared to ACA-lcSSc, with lower 10-year mortality than ATA-dcSSc. Presence of ACA in SSc has also been associated with a higher risk of ischemic macrovascular events, suggesting that autoantibodies may contribute directly to cardiovascular complications.^28^

In our cohort, none of the patients with limited or diffuse systemic sclerosis met the FiRST criteria for fibromyalgia, which is lower than the prevalence reported in previous studies. In a small study from India, Ayan et al.^8^ found a significantly higher prevalence of 62.79% in diffuse SSc patients using the 2010 FMS criteria compared to healthy controls. Differences in diagnostic criteria likely contributed to the variation in fibromyalgia prevalence between our study and others. The FiRST questionnaire was applied in our study using a cutoff of ≥5/6. While practical, it has shown lower specificity in connective tissue disease (CTD) populations compared to other groups; for instance, its specificity in CTD patients is reported at 59.6%, markedly lower than in rheumatoid arthritis or spondyloarthritis populations.^15^ In contrast, the 2010 American College of Rheumatology (ACR) initial criteria, often used in other studies, show higher specificity and sensitivity, especially with a modified cutoff score ≥13 (specificity 91.7%, sensitivity 93.1%).^66^ This variability in diagnostic tools and thresholds may explain the lower FM prevalence observed in our cohort.

In contrast, cross-sectional studies estimate the prevalence of depression in systemic sclerosis to range from 35% to 65%.^67^ In our cohort, only one patient reported mild depression, which may reflect underreporting due to the stigma surrounding mental health in the Middle East.^68^ These findings should be interpreted cautiously, as screening tools may underestimate psychological and somatic symptom burden, and cultural factors may influence reporting.

In our cohort, we did not see the expected clear separation in skin scores between diffuse and limited SSc. Many patients in both groups had only mild skin involvement (43.9% of limited vs. 26.3% of diffuse), and only a small number had severe disease (3.5% of limited vs. 5.3% of diffuse). Because of this overlap, the median skin scores ended up being closer than anticipated (median mRSS 8 [4–14] for limited vs. 14 [6.25–18] for diffuse). For comparison, a recent study from Bangladesh reported much higher scores overall, with a median mRSS of 19.0 (IQR 10–32), including 20.0 (15–34) for diffuse and 10.0 (6–21) for limited SSc.^22^ The smaller difference observed in our cohort may reflect milder disease at presentation, earlier diagnosis, or differences in referral patterns. Differences in disease duration and the variable course of skin thickening in SSc, where diffuse disease can regress over time,^69^ may also help explain why the two groups appeared more similar in our study.

**Figure 1.**
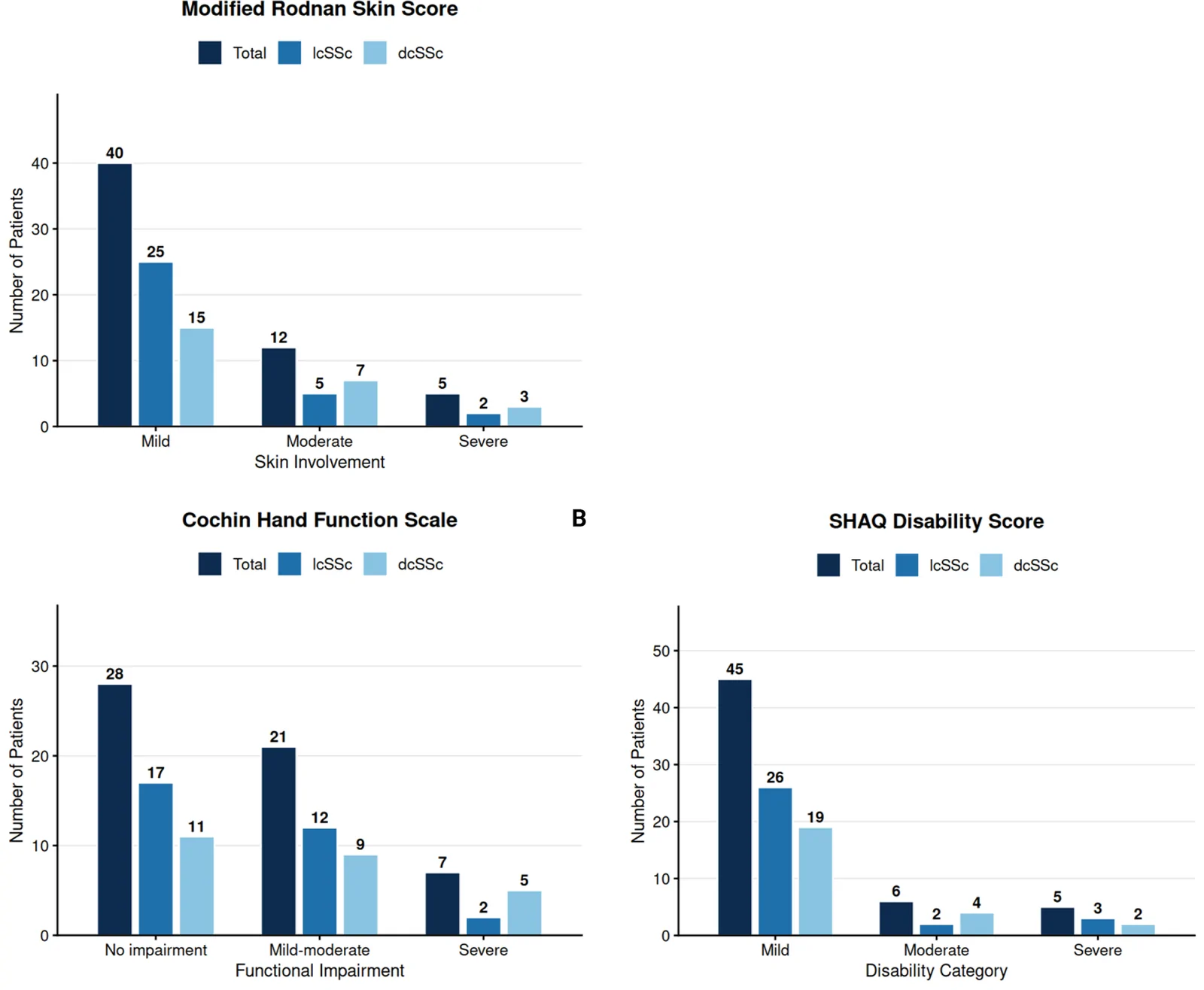
Comparison of the limited versus diffuse systemic sclerosis. **A:** Rodnan score; **B:** Cochin Hand Function; **C:** SHAQ score.

As shown in **Figure 1B**, half of the patients in our cohort (50%) reported no functional impairment, 37.5% experienced mild to moderate impairment, and 12.5% had severe functional limitations. The distribution of hand function impairment across lcSSc and dcSSc subtypes was relatively similar, with the dcSSc group exhibiting slightly more severe functional impairment, which is consistent with the findings of Brower et al.,^70^ who reported that individuals with dcSSc tended to have more significant hand function impairment. Despite differences in methodology, these findings highlight the functional challenges faced by patients with SSc, especially in the dcSSc subtype, where hand function impairment is more pronounced. A longitudinal study on hand disability in systemic sclerosis showed that hand function tends to worsen over time, especially in those who start with mild symptoms; recovery is rare, and once disability becomes severe, it is mostly irreversible within two years.^71^ A strong association was established between the Cochin Hand Function Scale and the SHAQ, both of which are self-reported disability scales.^70^

**Figure 2.**
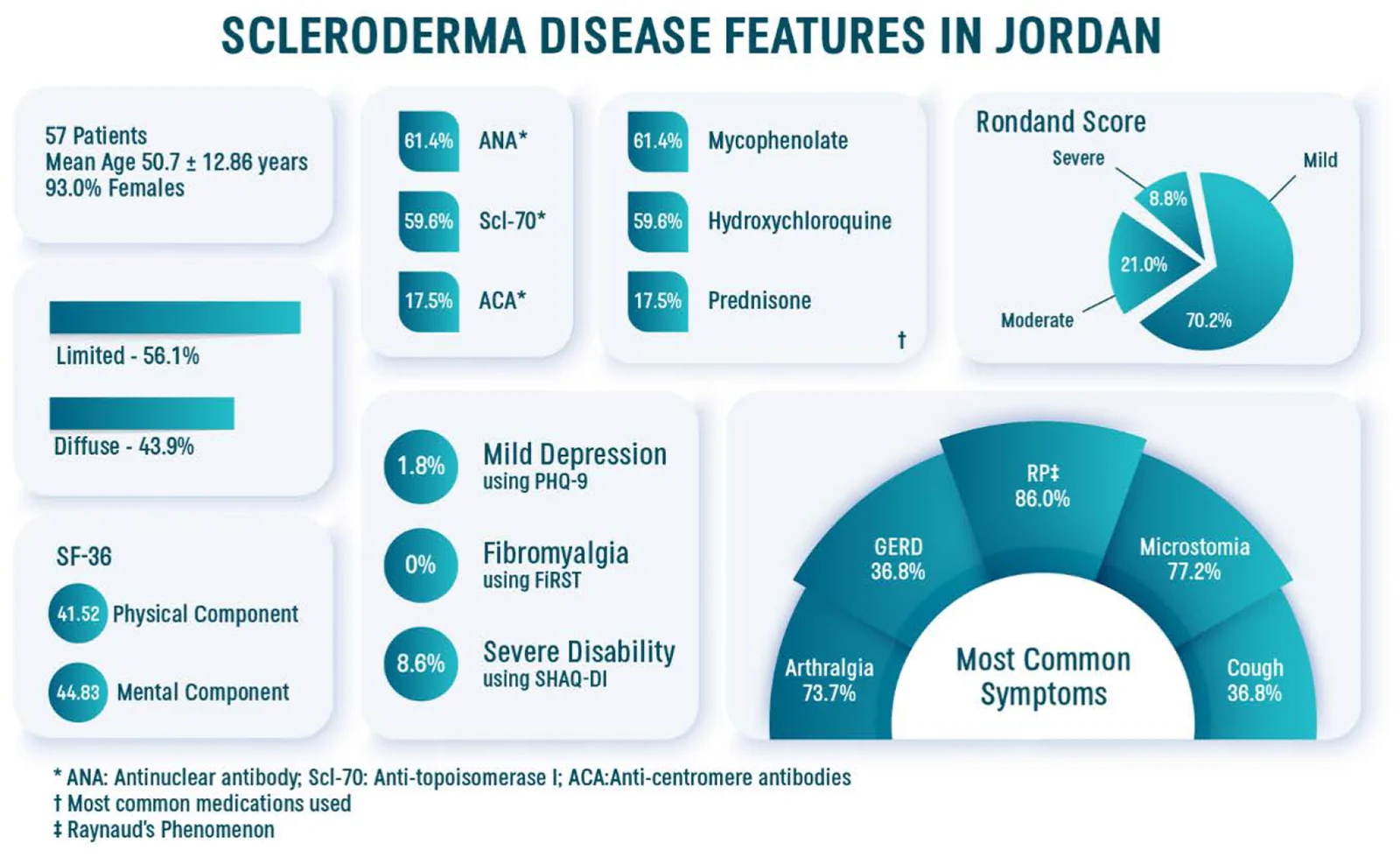
Infographic showing key clinical, serological, and quality of life features of systemic sclerosis in Jordan.

In our cohort, the overall SHAQ-DI median score was 0.25 (IQR: 0–1), with severe disability reported by only a small proportion of participants (8.9%). The distribution of disability severity revealed that the majority of limited SSc patients experienced mild disability (80.4%), while fewer dSSc patients fell into this category (46.5%) (**Figure 1C**). Moderate and severe disabilities were more pronounced in the diffuse subtype but remained relatively low across both subtypes. These findings are in contrast to previous studies, such as Brower et al.,^70^ which reported a higher SHAQ score of 1.03 ± 0.69 in their cohort of 40 scleroderma patients, and Alian et al.,^23^ who found a score of 1.34 ± 0.54 in 56 patients. Our cohort’s lower SHAQ-DI scores may reflect differences in disease management, duration, and population characteristics.

In terms of quality of life, evaluated using the SF-36, patients with the limited subtype had a median score of 90 (IQR: 40–100), while those with the diffuse subtype had a median score of 85 (IQR: 52.5–100). There were no statistically significant differences between the median or mean SF-36 scores of the two subtypes (p > 0.05). The mean general health score was 55.48 ± 24.78 for patients with limited disease and 65.00 ± 21.82 for those with diffuse disease. The SF-36 physical and mental component scores in our cohort were 41.52 [29.61–43.62] and 44.83 ± 10.39, respectively. These findings aligned with previous reports showing lower quality-of-life scores in systemic sclerosis (SSc) patients compared to the general population. A recent meta-analysis^72^ also reported pooled PCS and MCS means in SSc patients ranging from 31.20 to 52.80 and 37.40 to 68.30, respectively, confirming the consistent trend of reduced quality of life in this population. Notably, physical health tends to be more impaired than mental health in SSc patients, a trend that our findings also support.

## LIMITATIONS

The study has some limitations, including its single-centre design, relatively small sample size, and limited study duration, which may influence the generalisability of the findings. Some characteristics of our cohort, such as the comparatively lower prevalence of anticentromere antibodies, Raynaud’s phenomenon, gastrointestinal involvement, and ILD compared with international registries, may reflect local referral patterns, the spectrum of disease encountered at our centre, or natural variation in patient populations. These differences should be considered when interpreting the results in a broader context. As a cross-sectional analysis, the study was not designed to assess disease progression or long-term outcomes, and data on treatment response were not included. Future prospective, multicentre studies with larger cohorts and longitudinal follow-up would help further validate and expand upon these findings and provide additional insights into disease patterns and outcomes in the Jordanian population.

## CONCLUSIONS

This exploratory study provides preliminary descriptive data on systemic sclerosis in a Jordanian cohort, highlighting both the clinical features and the daily challenges faced by patients. While many individuals experienced only mild functional impairment, the burden of symptoms remains significant. Our findings emphasise the importance of early recognition, tailored treatment, and supportive care to help patients maintain quality of life. Future multicentre studies incorporating longitudinal follow-up, treatment response data, and standardised patient-reported outcomes are needed to better characterise the full disease burden of SSc across Jordan and the broader Arab region.

## Data Availability

The datasets generated and analysed during the current study are available from the corresponding author upon reasonable request, in accordance with institutional and ethical guidelines to protect patient confidentiality.
